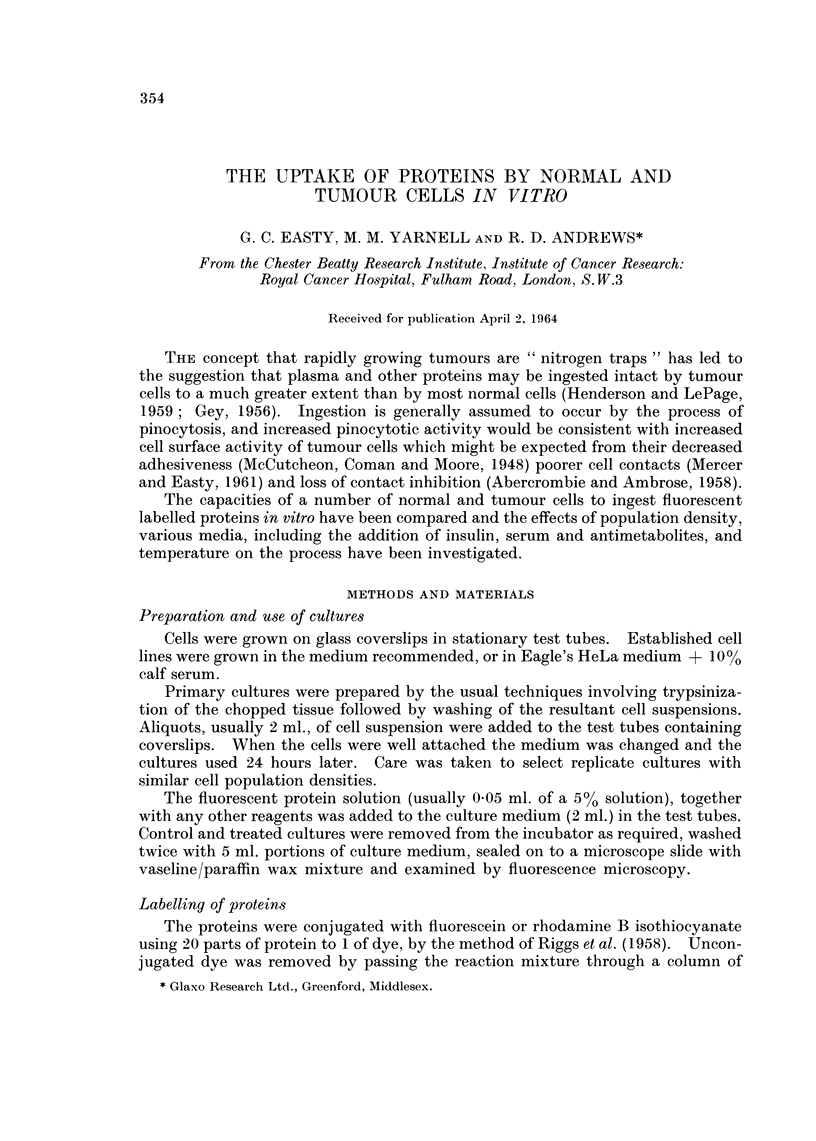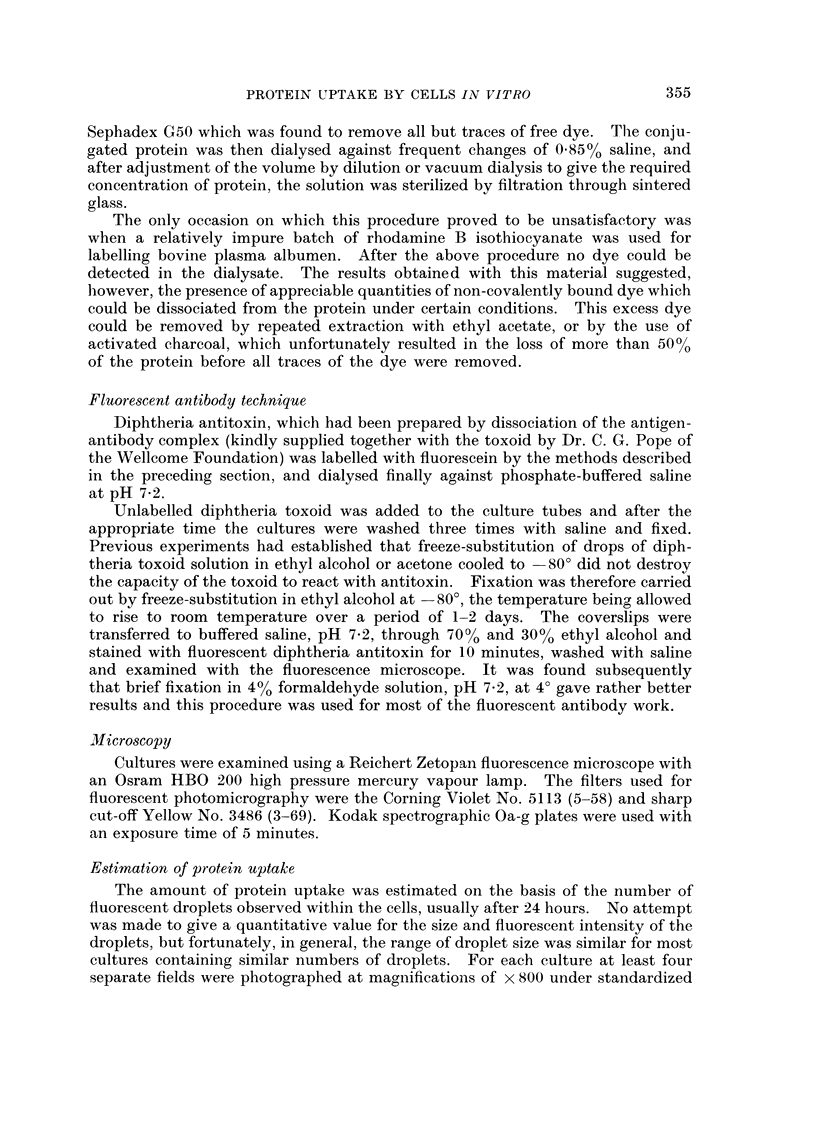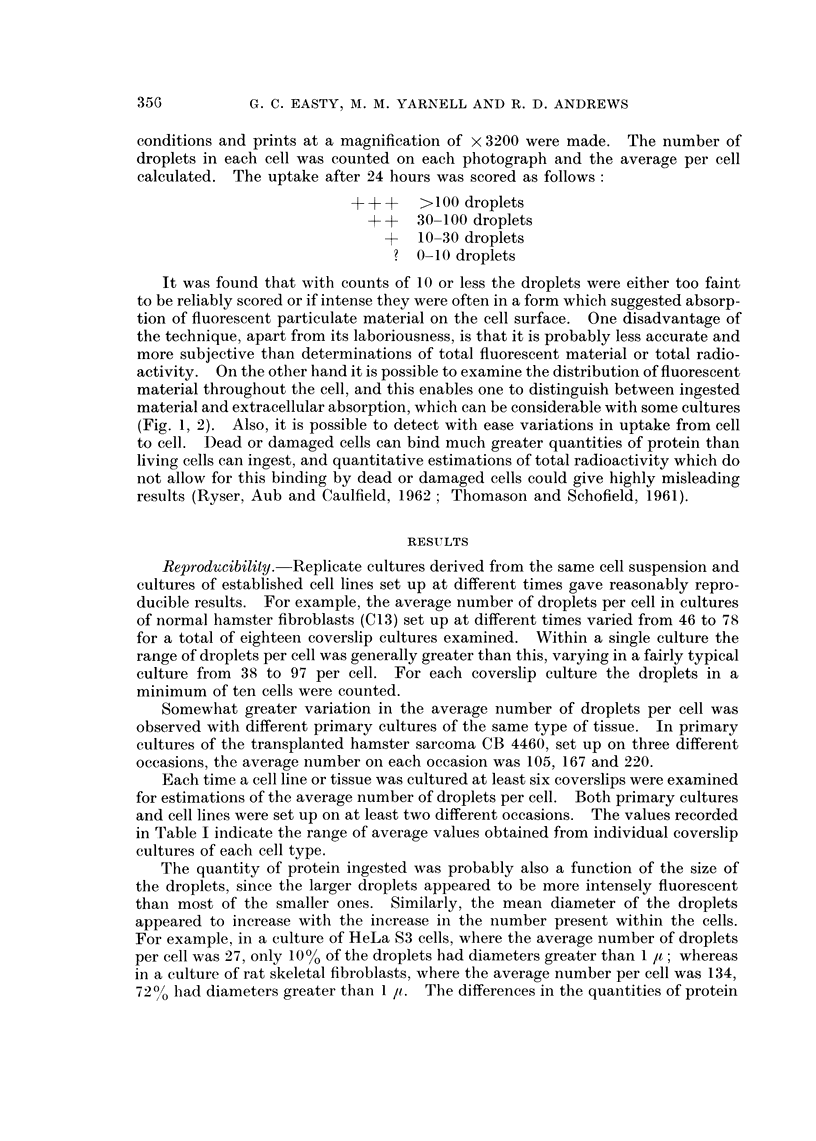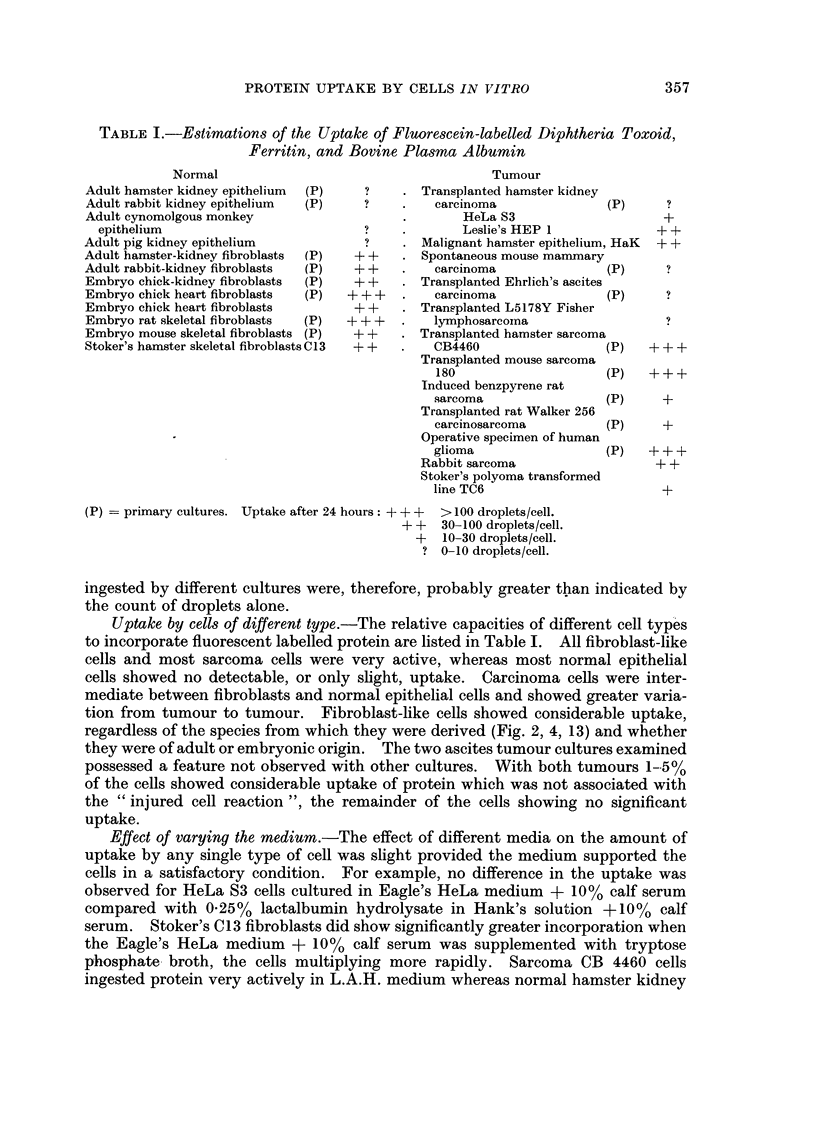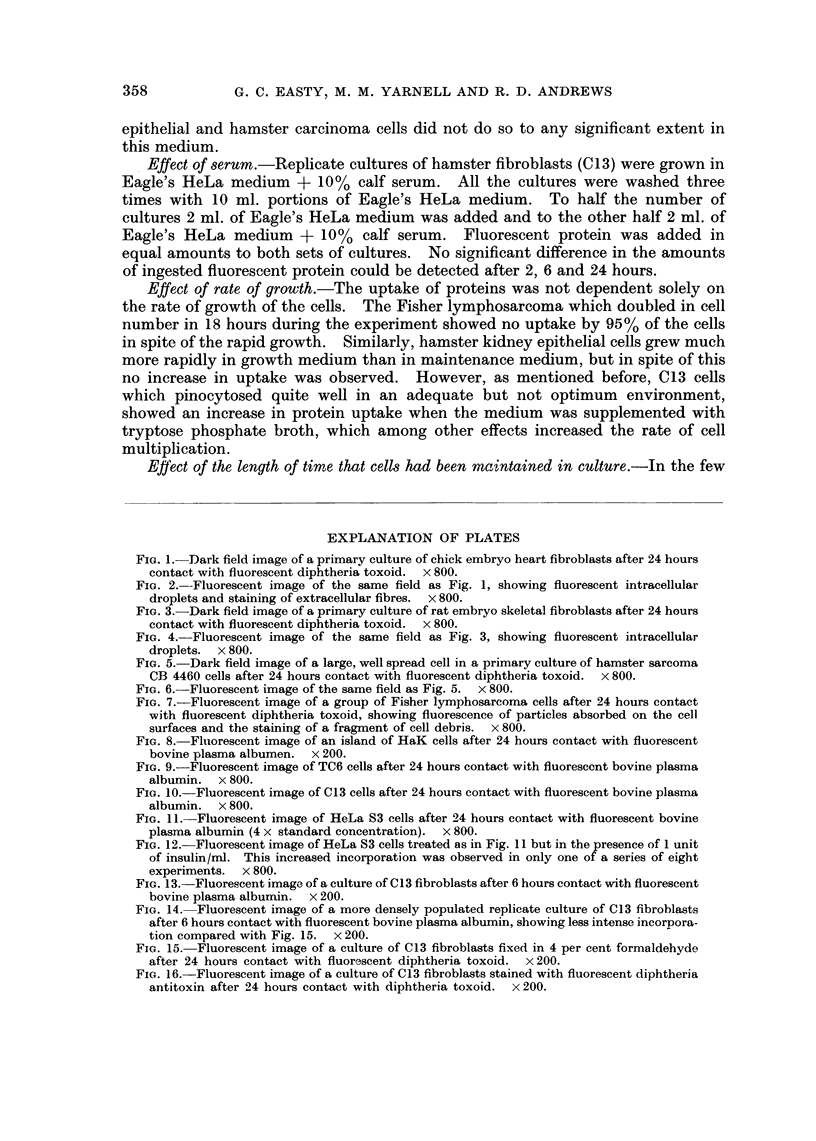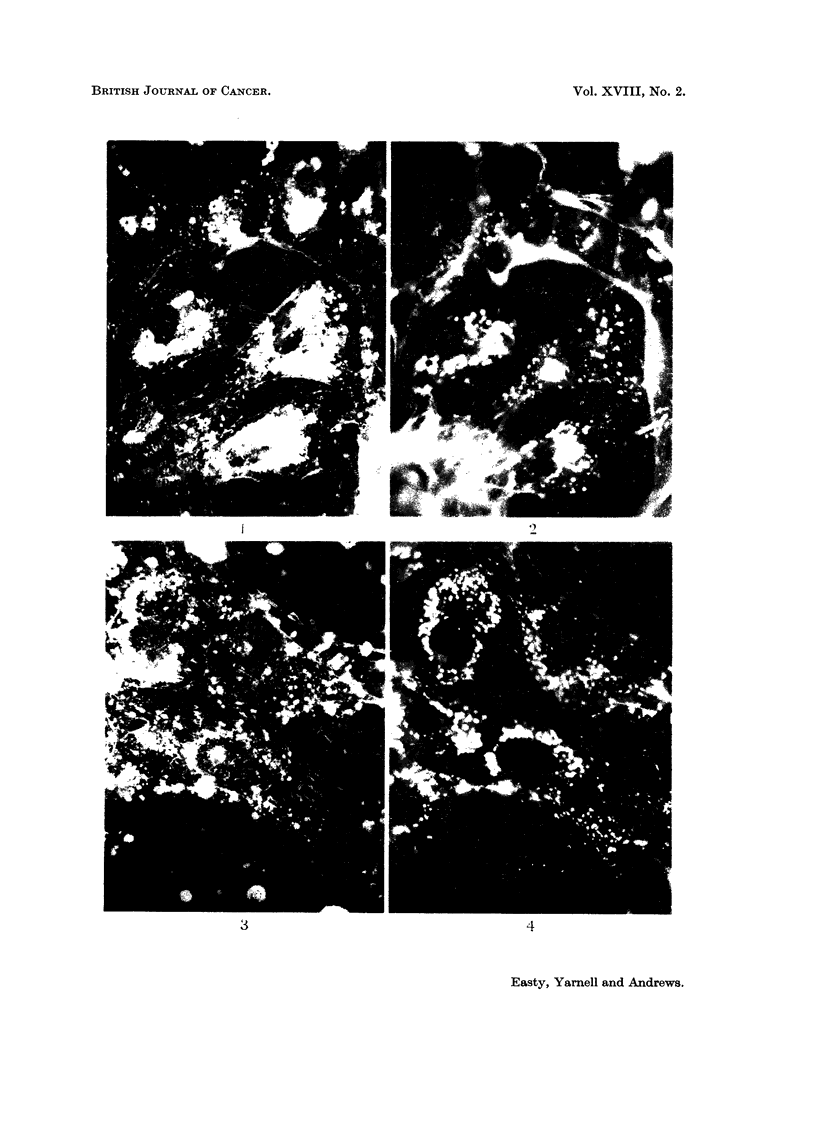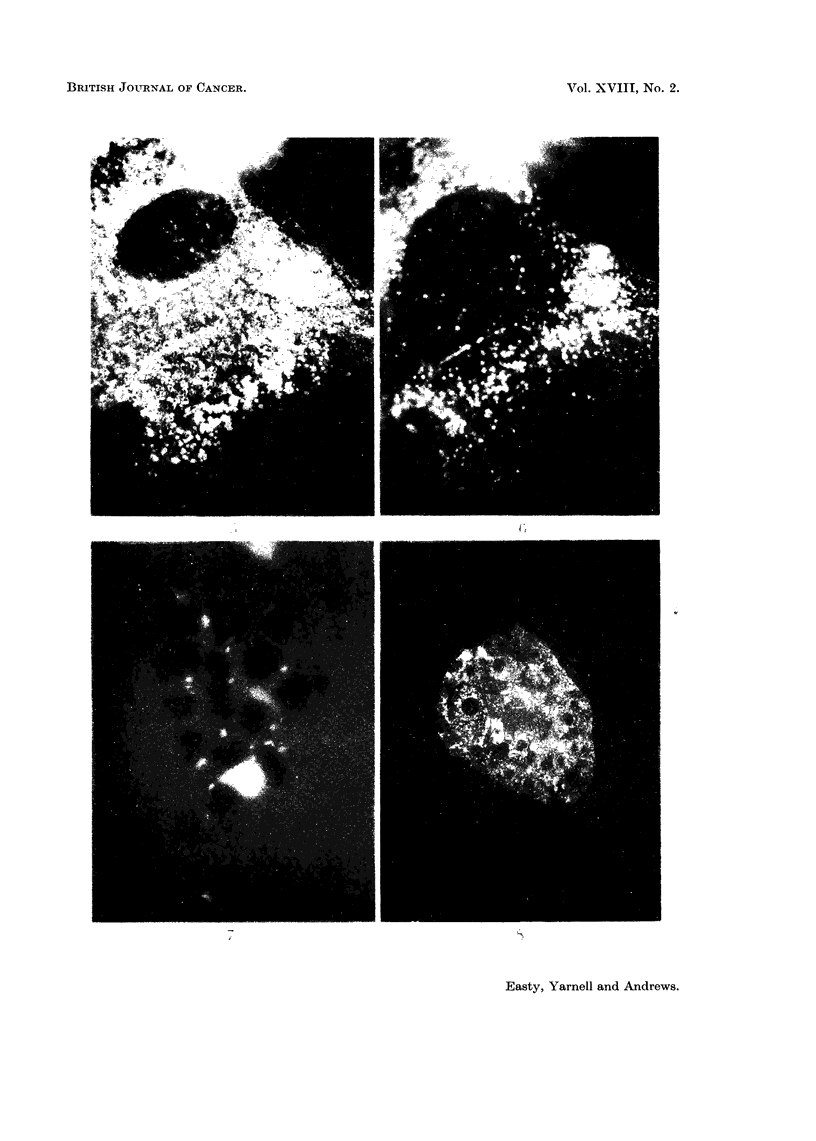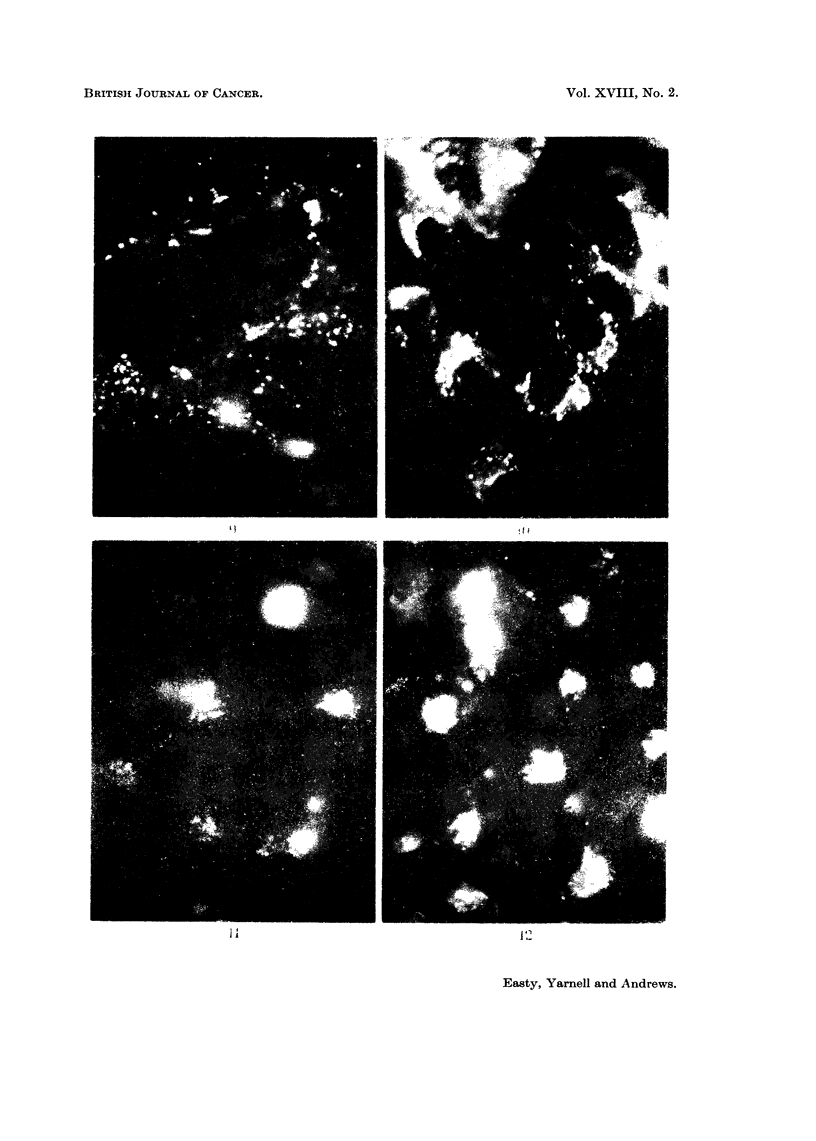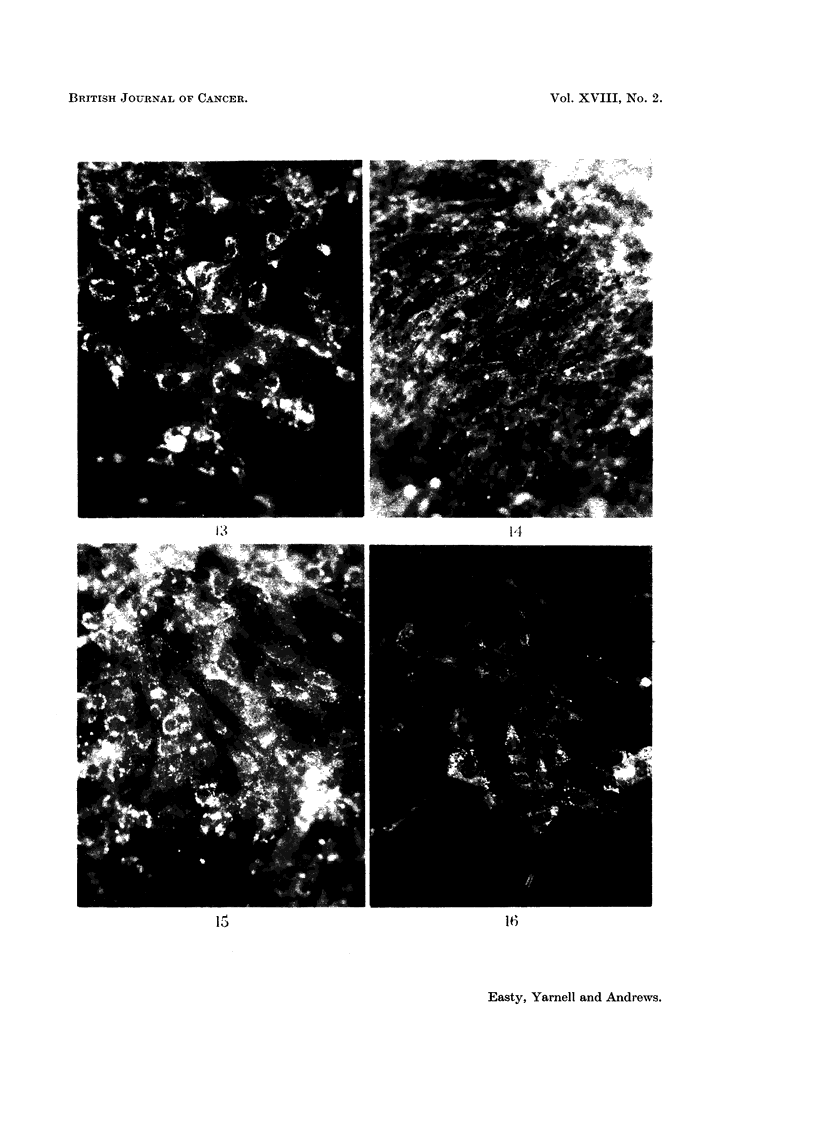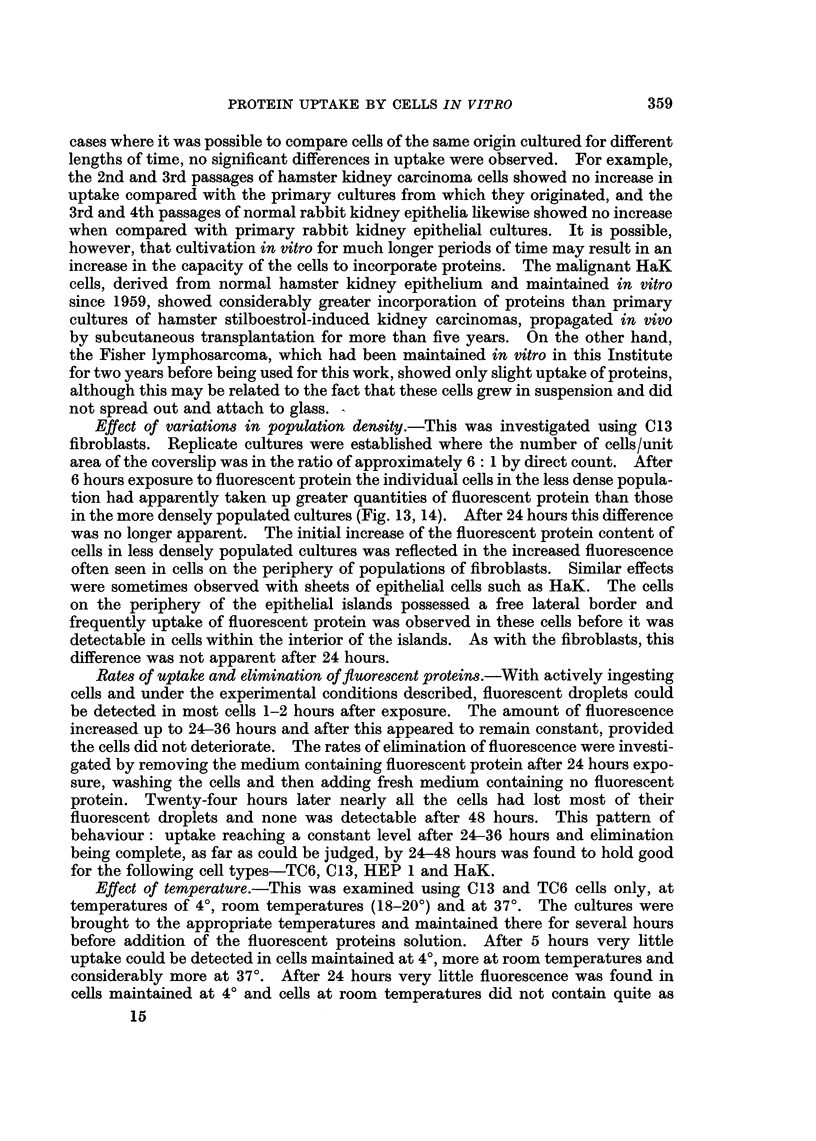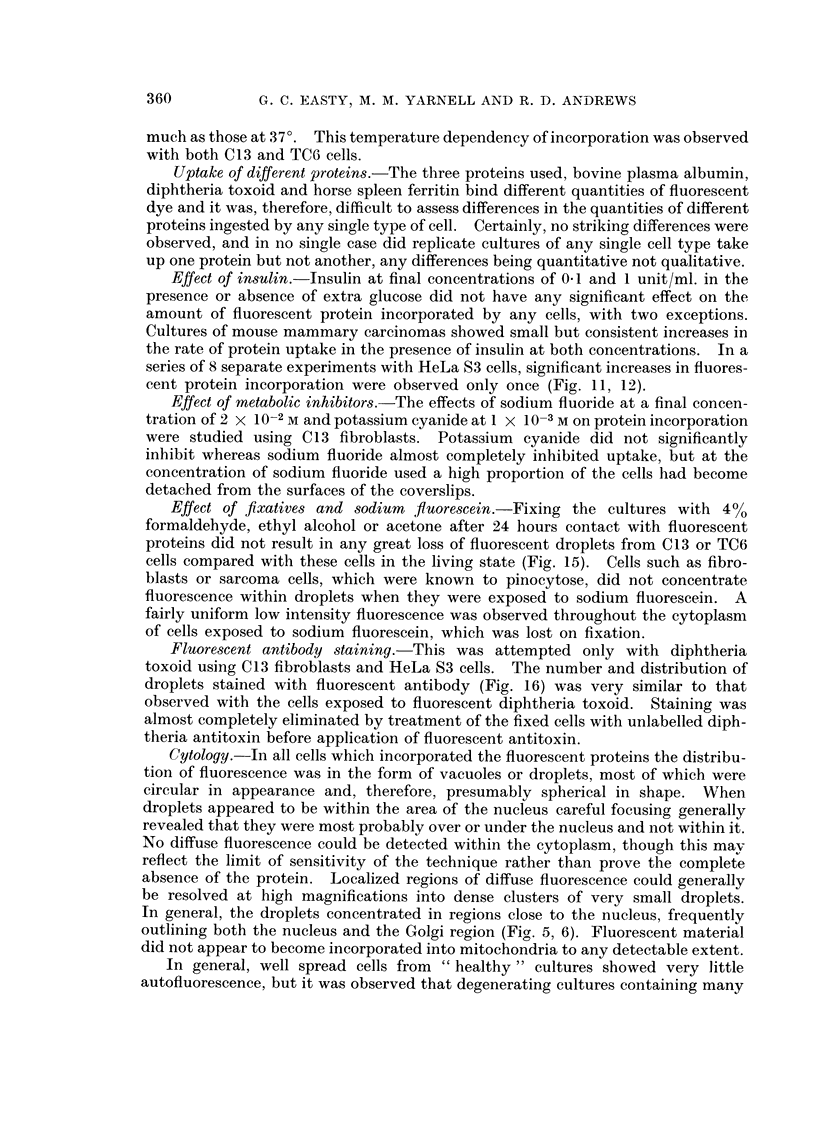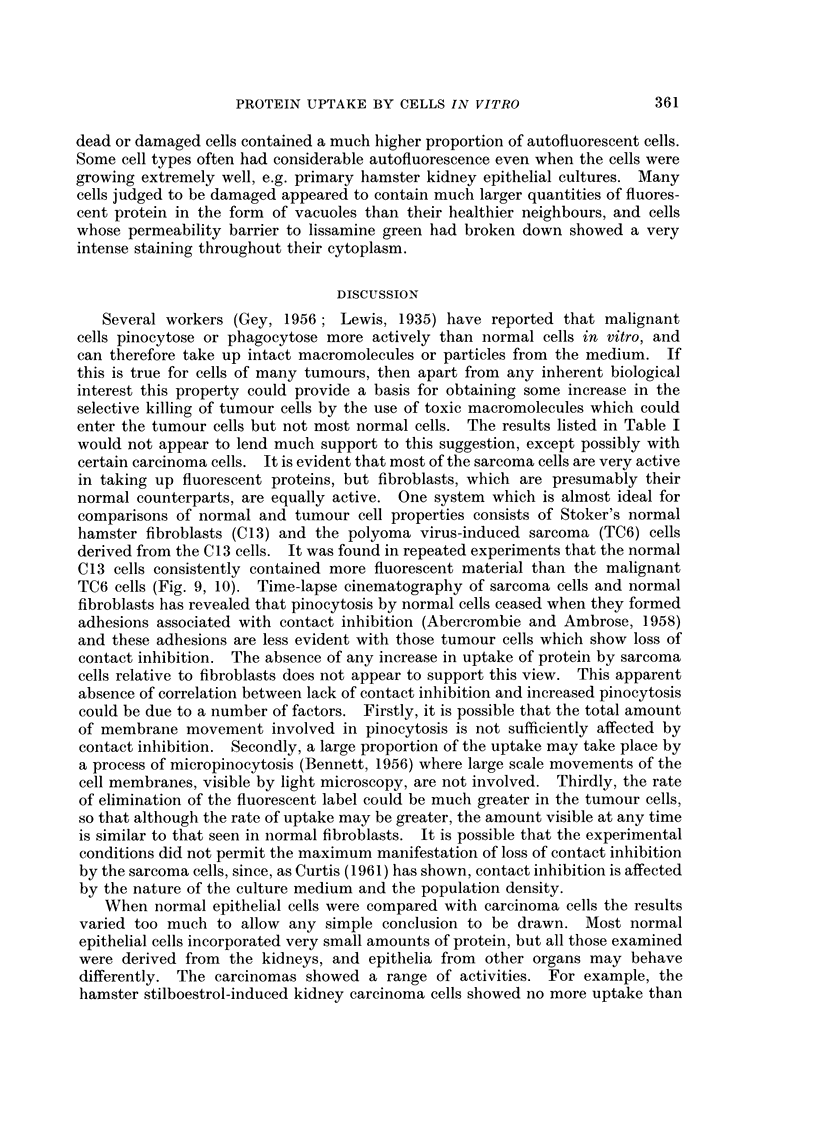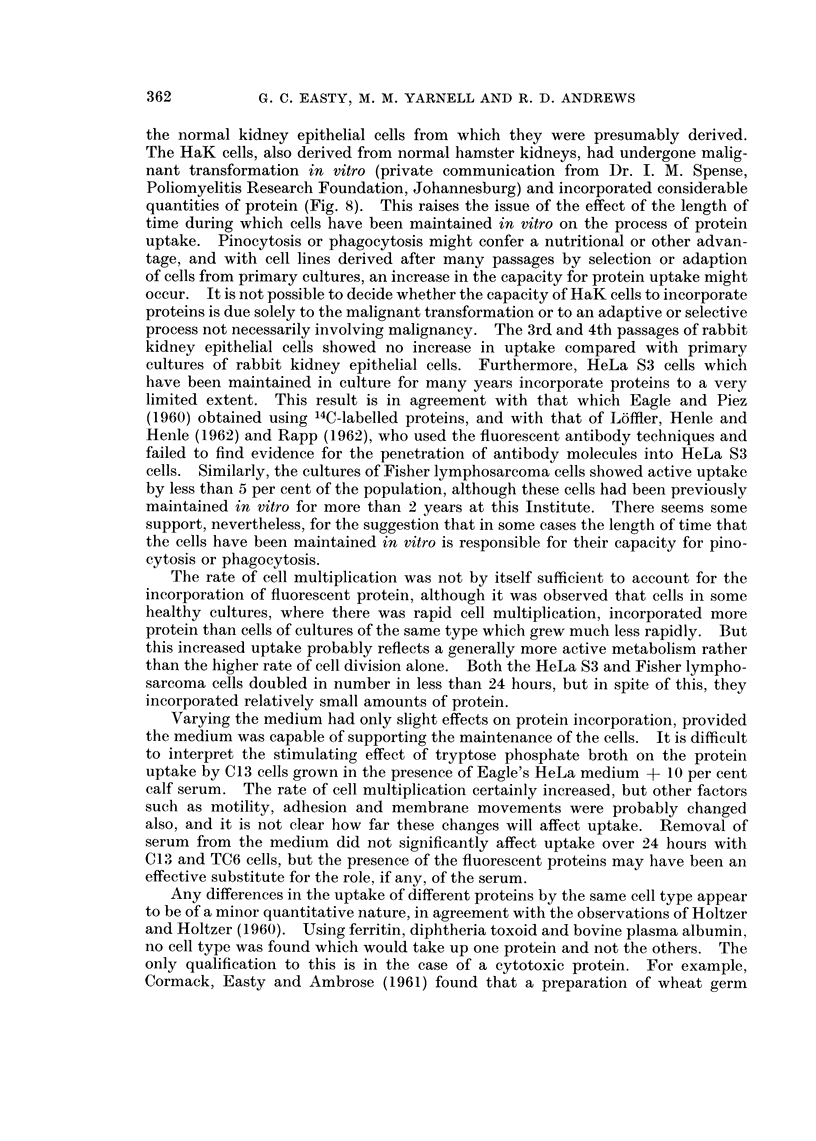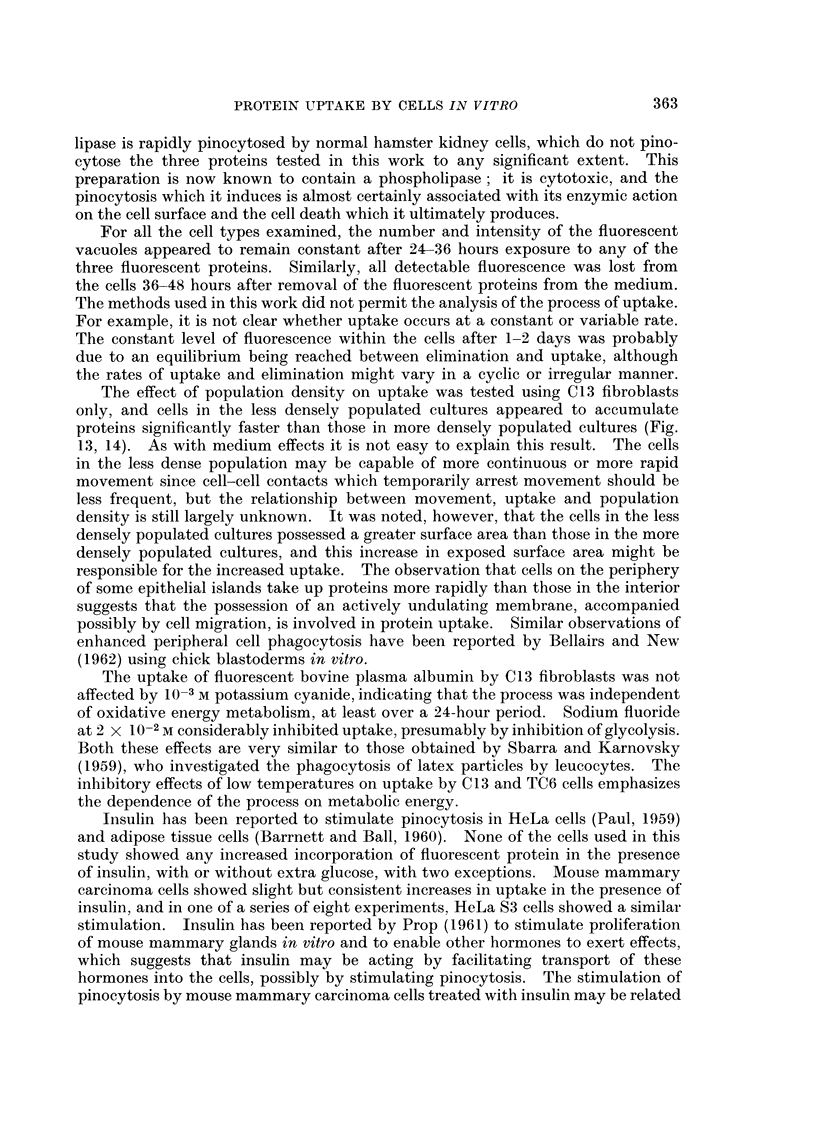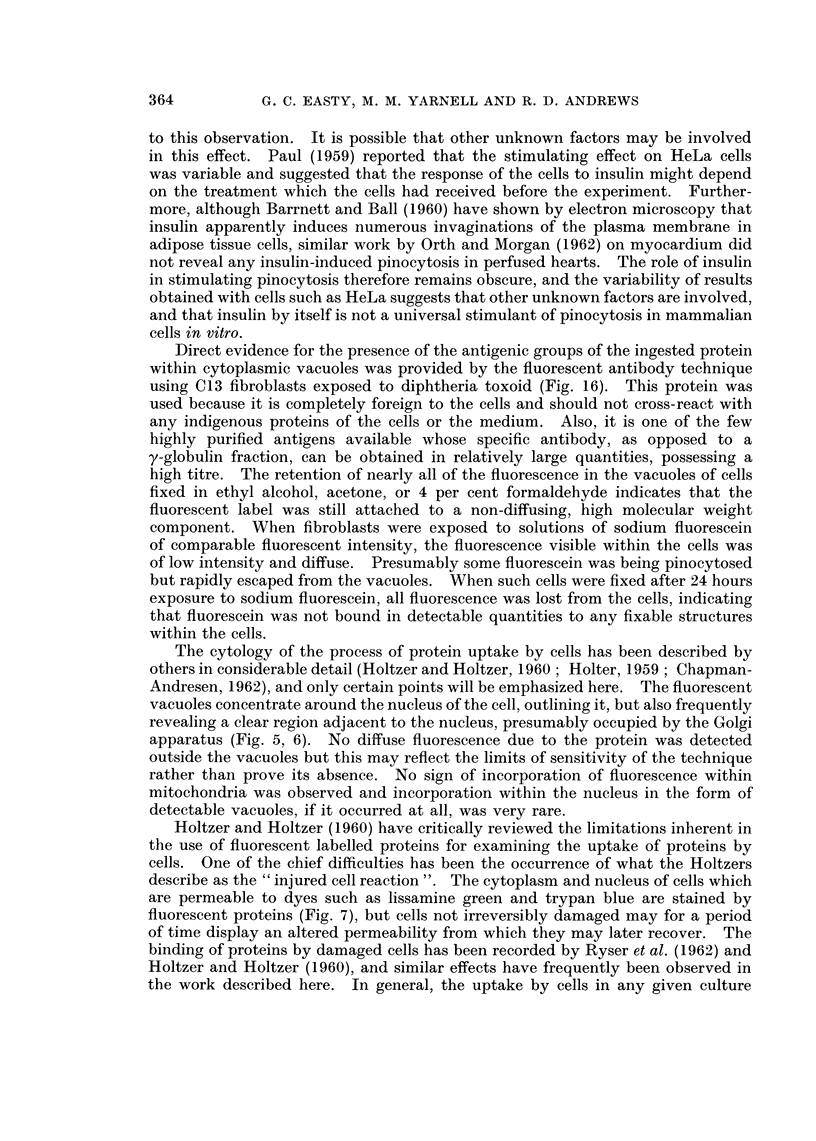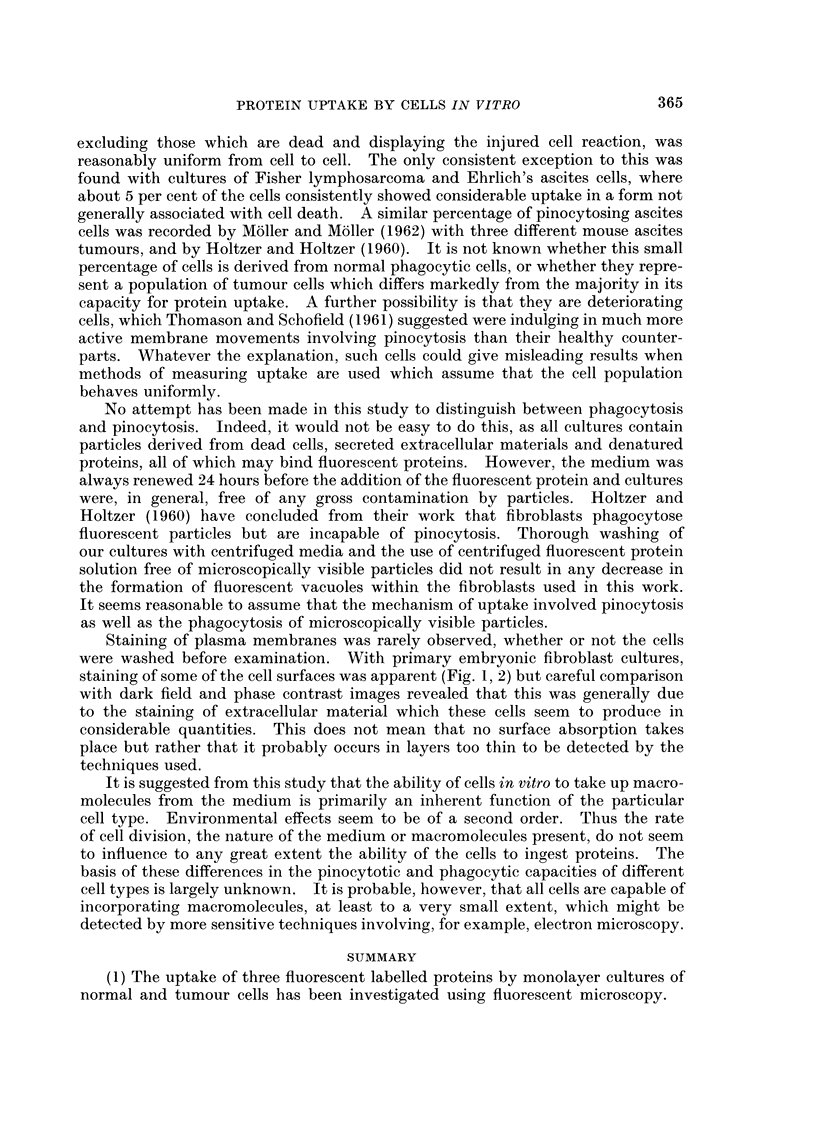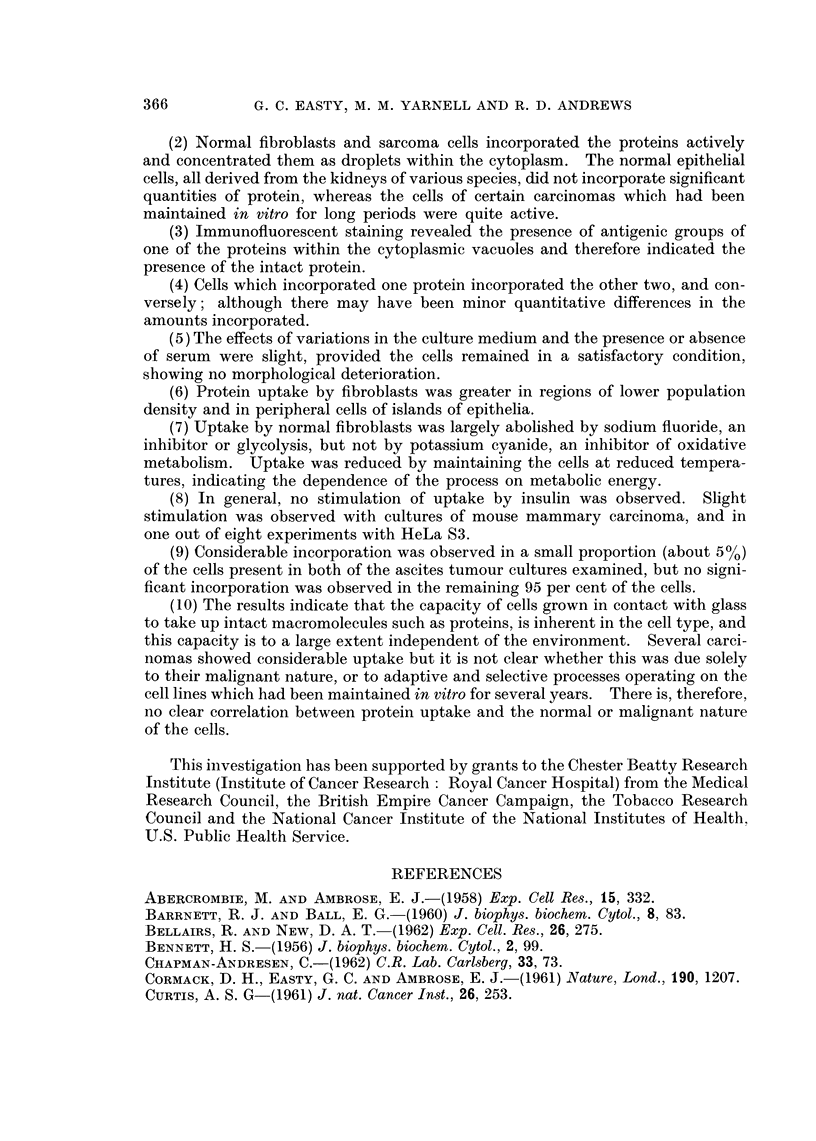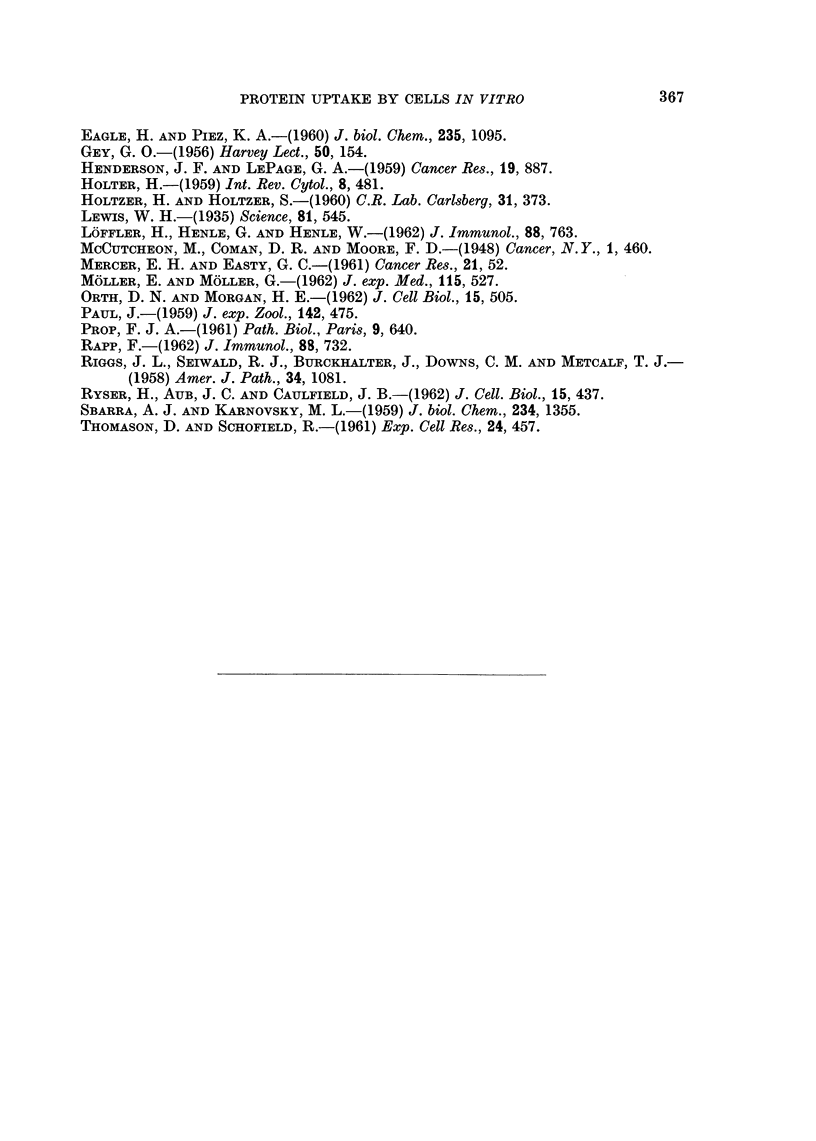# The Uptake of Proteins by Normal and Tumour Cells In Vitro

**DOI:** 10.1038/bjc.1964.41

**Published:** 1964-06

**Authors:** G. C. Easty, M. M. Yarnell, R. D. Andrews

## Abstract

**Images:**


### THE UPTAKE OF PROTEINS BY NORMAL AND TUMOUR CELLS *IN VITRO*

G. C. EASTY, M. M. YARNELL AND R. D. ANDREWS*

*From the Chester Beatty Research Institute, Institute of Cancer Research: Royal Cancer Hospital, Fulham Road, London, S.W.3*

Received for publication April 2, 1964

THE concept that rapidly growing tumours are " nitrogen traps " has led to the suggestion that plasma and other proteins may be ingested intact by tumour cells to a much greater extent than by most normal cells (Henderson and LePage, 1959 ; Gey, 1956). Ingestion is generally assumed to occur by the process of pinocytosis, and increased pinocytotic activity would be consistent with increased cell surface activity of tumour cells which might be expected from their decreased adhesiveness (McCutcheon, Coman and Moore, 1948) poorer cell contacts (Mercer and Easty, 1961) and loss of contact inhibition (Abercrombie and Ambrose, 1958).

The capacities of a number of normal and tumour cells to ingest fluorescent labelled proteins *in vitro* have been compared and the effects of population density, various media, including the addition of insulin, serum and antimetabolites, and temperature on the process have been investigated.

### METHODS AND MATERIALS

### *Preparation and use of cultures*

Cells were grown on glass coverslips in stationary test tubes. Established cell lines were grown in the medium recommended, or in Eagle's HeLa medium + 10% calf serum.

Primary cultures were prepared by the usual techniques involving trypsinization of the chopped tissue followed by washing of the resultant cell suspensions. Aliquots, usually 2 ml., of cell suspension were added to the test tubes containing coverslips. When the cells were well attached the medium was changed and the cultures used 24 hours later. Care was taken to select replicate cultures with similar cell population densities.

The fluorescent protein solution (usually 0·05 ml. of a 5% solution), together with any other reagents was added to the culture medium (2 ml.) in the test tubes. Control and treated cultures were removed from the incubator as required, washed twice with 5 ml. portions of culture medium, sealed on to a microscope slide with vaseline/paraffin wax mixture and examined by fluorescence microscopy.

### *Labelling of proteins*

The proteins were conjugated with fluorescein or rhodamine B isothiocyanate using 20 parts of protein to 1 of dye, by the method of Riggs *et al.* (1958). Unconjugated dye was removed by passing the reaction mixture through a column of

* Glaxo Research Ltd., Greenford, Middlesex.

Sephadex G50 which was found to remove all but traces of free dye. The conjugated protein was then dialysed against frequent changes of 0·85% saline, and after adjustment of the volume by dilution or vacuum dialysis to give the required concentration of protein, the solution was sterilized by filtration through sintered glass.

The only occasion on which this procedure proved to be unsatisfactory was when a relatively impure batch of rhodamine B isothiocyanate was used for labelling bovine plasma albumen. After the above procedure no dye could be detected in the dialysate. The results obtained with this material suggested, however, the presence of appreciable quantities of non-covalently bound dye which could be dissociated from the protein under certain conditions. This excess dye could be removed by repeated extraction with ethyl acetate, or by the use of activated charcoal, which unfortunately resulted in the loss of more than 50% of the protein before all traces of the dye were removed.

*Fluorescent antibody technique*

Diphtheria antitoxin, which had been prepared by dissociation of the antigen-antibody complex (kindly supplied together with the toxoid by Dr. C. G. Pope of the Wellcome Foundation) was labelled with fluorescein by the methods described in the preceding section, and dialysed finally against phosphate-buffered saline at pH 7·2.

Unlabelled diphtheria toxoid was added to the culture tubes and after the appropriate time the cultures were washed three times with saline and fixed. Previous experiments had established that freeze-substitution of drops of diphtheria toxoid solution in ethyl alcohol or acetone cooled to −80° did not destroy the capacity of the toxoid to react with antitoxin. Fixation was therefore carried out by freeze-substitution in ethyl alcohol at −80°, the temperature being allowed to rise to room temperature over a period of 1–2 days. The coverslips were transferred to buffered saline, pH 7·2, through 70% and 30% ethyl alcohol and stained with fluorescent diphtheria antitoxin for 10 minutes, washed with saline and examined with the fluorescence microscope. It was found subsequently that brief fixation in 4% formaldehyde solution, pH 7·2, at 4° gave rather better results and this procedure was used for most of the fluorescent antibody work.

*Microscopy*

Cultures were examined using a Reichert Zetopan fluorescence microscope with an Osram HBO 200 high pressure mercury vapour lamp. The filters used for fluorescent photomicrography were the Corning Violet No. 5113 (5–58) and sharp cut-off Yellow No. 3486 (3–69). Kodak spectrographic Oa-g plates were used with an exposure time of 5 minutes.

*Estimation of protein uptake*

The amount of protein uptake was estimated on the basis of the number of fluorescent droplets observed within the cells, usually after 24 hours. No attempt was made to give a quantitative value for the size and fluorescent intensity of the droplets, but fortunately, in general, the range of droplet size was similar for most cultures containing similar numbers of droplets. For each culture at least four separate fields were photographed at magnifications of ×800 under standardized conditions and prints at a magnification of $\times 3200$ were made. The number of droplets in each cell was counted on each photograph and the average per cell calculated. The uptake after 24 hours was scored as follows :

| | |
|---|---|
| +++ | >100 droplets |
| ++ | 30–100 droplets |
| + | 10–30 droplets |
| ? | 0–10 droplets |

It was found that with counts of 10 or less the droplets were either too faint to be reliably scored or if intense they were often in a form which suggested absorption of fluorescent particulate material on the cell surface. One disadvantage of the technique, apart from its laboriousness, is that it is probably less accurate and more subjective than determinations of total fluorescent material or total radioactivity. On the other hand it is possible to examine the distribution of fluorescent material throughout the cell, and this enables one to distinguish between ingested material and extracellular absorption, which can be considerable with some cultures (Fig. 1, 2). Also, it is possible to detect with ease variations in uptake from cell to cell. Dead or damaged cells can bind much greater quantities of protein than living cells can ingest, and quantitative estimations of total radioactivity which do not allow for this binding by dead or damaged cells could give highly misleading results (Ryser, Aub and Caulfield, 1962 ; Thomason and Schofield, 1961).

### RESULTS

*Reproducibility.*—Replicate cultures derived from the same cell suspension and cultures of established cell lines set up at different times gave reasonably reproducible results. For example, the average number of droplets per cell in cultures of normal hamster fibroblasts (C13) set up at different times varied from 46 to 78 for a total of eighteen coverslip cultures examined. Within a single culture the range of droplets per cell was generally greater than this, varying in a fairly typical culture from 38 to 97 per cell. For each coverslip culture the droplets in a minimum of ten cells were counted.

Somewhat greater variation in the average number of droplets per cell was observed with different primary cultures of the same type of tissue. In primary cultures of the transplanted hamster sarcoma CB 4460, set up on three different occasions, the average number on each occasion was 105, 167 and 220.

Each time a cell line or tissue was cultured at least six coverslips were examined for estimations of the average number of droplets per cell. Both primary cultures and cell lines were set up on at least two different occasions. The values recorded in Table I indicate the range of average values obtained from individual coverslip cultures of each cell type.

The quantity of protein ingested was probably also a function of the size of the droplets, since the larger droplets appeared to be more intensely fluorescent than most of the smaller ones. Similarly, the mean diameter of the droplets appeared to increase with the increase in the number present within the cells. For example, in a culture of HeLa S3 cells, where the average number of droplets per cell was 27, only 10% of the droplets had diameters greater than 1 $\mu$ ; whereas in a culture of rat skeletal fibroblasts, where the average number per cell was 134, 72% had diameters greater than 1 $\mu$. The differences in the quantities of protein

TABLE I.—*Estimations of the Uptake of Fluorescein-labelled Diphtheria Toxoid, Ferritin, and Bovine Plasma Albumin*

| Normal | | |
|---|---|---|
| Adult hamster kidney epithelium | (P) | ? |
| Adult rabbit kidney epithelium | (P) | ? |
| Adult cynomolgous monkey epithelium | | ? |
| Adult pig kidney epithelium | | ? |
| Adult hamster-kidney fibroblasts | (P) | ++ |
| Adult rabbit-kidney fibroblasts | (P) | ++ |
| Embryo chick-kidney fibroblasts | (P) | ++ |
| Embryo chick heart fibroblasts | (P) | +++ |
| Embryo chick heart fibroblasts | | ++ |
| Embryo rat skeletal fibroblasts | (P) | +++ |
| Embryo mouse skeletal fibroblasts | (P) | ++ |
| Stoker's hamster skeletal fibroblasts | C13 | ++ |

| Tumour | | |
|---|---|---|
| Transplanted hamster kidney carcinoma | (P) | ? |
| HeLa S3 | | + |
| Leslie's HEP 1 | | ++ |
| Malignant hamster epithelium, HaK | | ++ |
| Spontaneous mouse mammary carcinoma | (P) | ? |
| Transplanted Ehrlich's ascites carcinoma | (P) | ? |
| Transplanted L5178Y Fisher lymphosarcoma | | ? |
| Transplanted hamster sarcoma CB4460 | (P) | +++ |
| Transplanted mouse sarcoma 180 | (P) | +++ |
| Induced benzpyrene rat sarcoma | (P) | + |
| Transplanted rat Walker 256 carcinosarcoma | (P) | + |
| Operative specimen of human glioma | (P) | +++ |
| Rabbit sarcoma | | ++ |
| Stoker's polyoma transformed line TC6 | | + |

(P) = primary cultures. Uptake after 24 hours : +++ >100 droplets/cell.
++ 30–100 droplets/cell.
+ 10–30 droplets/cell.
? 0–10 droplets/cell.

ingested by different cultures were, therefore, probably greater than indicated by the count of droplets alone.

*Uptake by cells of different type.*—The relative capacities of different cell types to incorporate fluorescent labelled protein are listed in Table I. All fibroblast-like cells and most sarcoma cells were very active, whereas most normal epithelial cells showed no detectable, or only slight, uptake. Carcinoma cells were intermediate between fibroblasts and normal epithelial cells and showed greater variation from tumour to tumour. Fibroblast-like cells showed considerable uptake, regardless of the species from which they were derived (Fig. 2, 4, 13) and whether they were of adult or embryonic origin. The two ascites tumour cultures examined possessed a feature not observed with other cultures. With both tumours 1–5% of the cells showed considerable uptake of protein which was not associated with the " injured cell reaction ", the remainder of the cells showing no significant uptake.

*Effect of varying the medium.*—The effect of different media on the amount of uptake by any single type of cell was slight provided the medium supported the cells in a satisfactory condition. For example, no difference in the uptake was observed for HeLa S3 cells cultured in Eagle's HeLa medium + 10% calf serum compared with 0·25% lactalbumin hydrolysate in Hank's solution +10% calf serum. Stoker's C13 fibroblasts did show significantly greater incorporation when the Eagle's HeLa medium + 10% calf serum was supplemented with tryptose phosphate broth, the cells multiplying more rapidly. Sarcoma CB 4460 cells ingested protein very actively in L.A.H. medium whereas normal hamster kidney epithelial and hamster carcinoma cells did not do so to any significant extent in this medium.

*Effect of serum.*—Replicate cultures of hamster fibroblasts (C13) were grown in Eagle's HeLa medium + 10% calf serum. All the cultures were washed three times with 10 ml. portions of Eagle's HeLa medium. To half the number of cultures 2 ml. of Eagle's HeLa medium was added and to the other half 2 ml. of Eagle's HeLa medium + 10% calf serum. Fluorescent protein was added in equal amounts to both sets of cultures. No significant difference in the amounts of ingested fluorescent protein could be detected after 2, 6 and 24 hours.

*Effect of rate of growth.*—The uptake of proteins was not dependent solely on the rate of growth of the cells. The Fisher lymphosarcoma which doubled in cell number in 18 hours during the experiment showed no uptake by 95% of the cells in spite of the rapid growth. Similarly, hamster kidney epithelial cells grew much more rapidly in growth medium than in maintenance medium, but in spite of this no increase in uptake was observed. However, as mentioned before, C13 cells which pinocytosed quite well in an adequate but not optimum environment, showed an increase in protein uptake when the medium was supplemented with tryptose phosphate broth, which among other effects increased the rate of cell multiplication.

*Effect of the length of time that cells had been maintained in culture.*—In the few

---

### EXPLANATION OF PLATES

FIG. 1.—Dark field image of a primary culture of chick embryo heart fibroblasts after 24 hours contact with fluorescent diphtheria toxoid. ×800.

FIG. 2.—Fluorescent image of the same field as Fig. 1, showing fluorescent intracellular droplets and staining of extracellular fibres. ×800.

FIG. 3.—Dark field image of a primary culture of rat embryo skeletal fibroblasts after 24 hours contact with fluorescent diphtheria toxoid. ×800.

FIG. 4.—Fluorescent image of the same field as Fig. 3, showing fluorescent intracellular droplets. ×800.

FIG. 5.—Dark field image of a large, well spread cell in a primary culture of hamster sarcoma CB 4460 cells after 24 hours contact with fluorescent diphtheria toxoid. ×800.

FIG. 6.—Fluorescent image of the same field as Fig. 5. ×800.

FIG. 7.—Fluorescent image of a group of Fisher lymphosarcoma cells after 24 hours contact with fluorescent diphtheria toxoid, showing fluorescence of particles absorbed on the cell surfaces and the staining of a fragment of cell debris. ×800.

FIG. 8.—Fluorescent image of an island of HaK cells after 24 hours contact with fluorescent bovine plasma albumen. ×200.

FIG. 9.—Fluorescent image of TC6 cells after 24 hours contact with fluorescent bovine plasma albumin. ×800.

FIG. 10.—Fluorescent image of C13 cells after 24 hours contact with fluorescent bovine plasma albumin. ×800.

FIG. 11.—Fluorescent image of HeLa S3 cells after 24 hours contact with fluorescent bovine plasma albumin (4× standard concentration). ×800.

FIG. 12.—Fluorescent image of HeLa S3 cells treated as in Fig. 11 but in the presence of 1 unit of insulin/ml. This increased incorporation was observed in only one of a series of eight experiments. ×800.

FIG. 13.—Fluorescent image of a culture of C13 fibroblasts after 6 hours contact with fluorescent bovine plasma albumin. ×200.

FIG. 14.—Fluorescent image of a more densely populated replicate culture of C13 fibroblasts after 6 hours contact with fluorescent bovine plasma albumin, showing less intense incorporation compared with Fig. 15. ×200.

FIG. 15.—Fluorescent image of a culture of C13 fibroblasts fixed in 4 per cent formaldehyde after 24 hours contact with fluorescent diphtheria toxoid. ×200.

FIG. 16.—Fluorescent image of a culture of C13 fibroblasts stained with fluorescent diphtheria antitoxin after 24 hours contact with diphtheria toxoid. ×200.

1

2

3

4

Easty, Yarnell and Andrews.

5

6

7

8

Easty, Yarnell and Andrews.

9

10

11

12

Easty, Yarnell and Andrews.

13

14

15

16

Easty, Yarnell and Andrews.

cases where it was possible to compare cells of the same origin cultured for different lengths of time, no significant differences in uptake were observed. For example, the 2nd and 3rd passages of hamster kidney carcinoma cells showed no increase in uptake compared with the primary cultures from which they originated, and the 3rd and 4th passages of normal rabbit kidney epithelia likewise showed no increase when compared with primary rabbit kidney epithelial cultures. It is possible, however, that cultivation *in vitro* for much longer periods of time may result in an increase in the capacity of the cells to incorporate proteins. The malignant HaK cells, derived from normal hamster kidney epithelium and maintained *in vitro* since 1959, showed considerably greater incorporation of proteins than primary cultures of hamster stilboestrol-induced kidney carcinomas, propagated *in vivo* by subcutaneous transplantation for more than five years. On the other hand, the Fisher lymphosarcoma, which had been maintained *in vitro* in this Institute for two years before being used for this work, showed only slight uptake of proteins, although this may be related to the fact that these cells grew in suspension and did not spread out and attach to glass.

*Effect of variations in population density.*—This was investigated using C13 fibroblasts. Replicate cultures were established where the number of cells/unit area of the coverslip was in the ratio of approximately 6 : 1 by direct count. After 6 hours exposure to fluorescent protein the individual cells in the less dense population had apparently taken up greater quantities of fluorescent protein than those in the more densely populated cultures (Fig. 13, 14). After 24 hours this difference was no longer apparent. The initial increase of the fluorescent protein content of cells in less densely populated cultures was reflected in the increased fluorescence often seen in cells on the periphery of populations of fibroblasts. Similar effects were sometimes observed with sheets of epithelial cells such as HaK. The cells on the periphery of the epithelial islands possessed a free lateral border and frequently uptake of fluorescent protein was observed in these cells before it was detectable in cells within the interior of the islands. As with the fibroblasts, this difference was not apparent after 24 hours.

*Rates of uptake and elimination of fluorescent proteins.*—With actively ingesting cells and under the experimental conditions described, fluorescent droplets could be detected in most cells 1–2 hours after exposure. The amount of fluorescence increased up to 24–36 hours and after this appeared to remain constant, provided the cells did not deteriorate. The rates of elimination of fluorescence were investigated by removing the medium containing fluorescent protein after 24 hours exposure, washing the cells and then adding fresh medium containing no fluorescent protein. Twenty-four hours later nearly all the cells had lost most of their fluorescent droplets and none was detectable after 48 hours. This pattern of behaviour : uptake reaching a constant level after 24–36 hours and elimination being complete, as far as could be judged, by 24–48 hours was found to hold good for the following cell types—TC6, C13, HEP 1 and HaK.

*Effect of temperature.*—This was examined using C13 and TC6 cells only, at temperatures of 4°, room temperatures (18–20°) and at 37°. The cultures were brought to the appropriate temperatures and maintained there for several hours before addition of the fluorescent proteins solution. After 5 hours very little uptake could be detected in cells maintained at 4°, more at room temperatures and considerably more at 37°. After 24 hours very little fluorescence was found in cells maintained at 4° and cells at room temperatures did not contain quite as much as those at 37°. This temperature dependency of incorporation was observed with both C13 and TC6 cells.

*Uptake of different proteins.*—The three proteins used, bovine plasma albumin, diphtheria toxoid and horse spleen ferritin bind different quantities of fluorescent dye and it was, therefore, difficult to assess differences in the quantities of different proteins ingested by any single type of cell. Certainly, no striking differences were observed, and in no single case did replicate cultures of any single cell type take up one protein but not another, any differences being quantitative not qualitative.

*Effect of insulin.*—Insulin at final concentrations of 0·1 and 1 unit/ml. in the presence or absence of extra glucose did not have any significant effect on the amount of fluorescent protein incorporated by any cells, with two exceptions. Cultures of mouse mammary carcinomas showed small but consistent increases in the rate of protein uptake in the presence of insulin at both concentrations. In a series of 8 separate experiments with HeLa S3 cells, significant increases in fluorescent protein incorporation were observed only once (Fig. 11, 12).

*Effect of metabolic inhibitors.*—The effects of sodium fluoride at a final concentration of $2 \times 10^{-2}$ M and potassium cyanide at $1 \times 10^{-3}$ M on protein incorporation were studied using C13 fibroblasts. Potassium cyanide did not significantly inhibit whereas sodium fluoride almost completely inhibited uptake, but at the concentration of sodium fluoride used a high proportion of the cells had become detached from the surfaces of the coverslips.

*Effect of fixatives and sodium fluorescein.*—Fixing the cultures with 4% formaldehyde, ethyl alcohol or acetone after 24 hours contact with fluorescent proteins did not result in any great loss of fluorescent droplets from C13 or TC6 cells compared with these cells in the living state (Fig. 15). Cells such as fibroblasts or sarcoma cells, which were known to pinocytose, did not concentrate fluorescence within droplets when they were exposed to sodium fluorescein. A fairly uniform low intensity fluorescence was observed throughout the cytoplasm of cells exposed to sodium fluorescein, which was lost on fixation.

*Fluorescent antibody staining.*—This was attempted only with diphtheria toxoid using C13 fibroblasts and HeLa S3 cells. The number and distribution of droplets stained with fluorescent antibody (Fig. 16) was very similar to that observed with the cells exposed to fluorescent diphtheria toxoid. Staining was almost completely eliminated by treatment of the fixed cells with unlabelled diphtheria antitoxin before application of fluorescent antitoxin.

*Cytology.*—In all cells which incorporated the fluorescent proteins the distribution of fluorescence was in the form of vacuoles or droplets, most of which were circular in appearance and, therefore, presumably spherical in shape. When droplets appeared to be within the area of the nucleus careful focusing generally revealed that they were most probably over or under the nucleus and not within it. No diffuse fluorescence could be detected within the cytoplasm, though this may reflect the limit of sensitivity of the technique rather than prove the complete absence of the protein. Localized regions of diffuse fluorescence could generally be resolved at high magnifications into dense clusters of very small droplets. In general, the droplets concentrated in regions close to the nucleus, frequently outlining both the nucleus and the Golgi region (Fig. 5, 6). Fluorescent material did not appear to become incorporated into mitochondria to any detectable extent.

In general, well spread cells from " healthy " cultures showed very little autofluorescence, but it was observed that degenerating cultures containing many dead or damaged cells contained a much higher proportion of autofluorescent cells. Some cell types often had considerable autofluorescence even when the cells were growing extremely well, e.g. primary hamster kidney epithelial cultures. Many cells judged to be damaged appeared to contain much larger quantities of fluorescent protein in the form of vacuoles than their healthier neighbours, and cells whose permeability barrier to lissamine green had broken down showed a very intense staining throughout their cytoplasm.

### DISCUSSION

Several workers (Gey, 1956; Lewis, 1935) have reported that malignant cells pinocytose or phagocytose more actively than normal cells *in vitro*, and can therefore take up intact macromolecules or particles from the medium. If this is true for cells of many tumours, then apart from any inherent biological interest this property could provide a basis for obtaining some increase in the selective killing of tumour cells by the use of toxic macromolecules which could enter the tumour cells but not most normal cells. The results listed in Table I would not appear to lend much support to this suggestion, except possibly with certain carcinoma cells. It is evident that most of the sarcoma cells are very active in taking up fluorescent proteins, but fibroblasts, which are presumably their normal counterparts, are equally active. One system which is almost ideal for comparisons of normal and tumour cell properties consists of Stoker's normal hamster fibroblasts (C13) and the polyoma virus-induced sarcoma (TC6) cells derived from the C13 cells. It was found in repeated experiments that the normal C13 cells consistently contained more fluorescent material than the malignant TC6 cells (Fig. 9, 10). Time-lapse cinematography of sarcoma cells and normal fibroblasts has revealed that pinocytosis by normal cells ceased when they formed adhesions associated with contact inhibition (Abercrombie and Ambrose, 1958) and these adhesions are less evident with those tumour cells which show loss of contact inhibition. The absence of any increase in uptake of protein by sarcoma cells relative to fibroblasts does not appear to support this view. This apparent absence of correlation between lack of contact inhibition and increased pinocytosis could be due to a number of factors. Firstly, it is possible that the total amount of membrane movement involved in pinocytosis is not sufficiently affected by contact inhibition. Secondly, a large proportion of the uptake may take place by a process of micropinocytosis (Bennett, 1956) where large scale movements of the cell membranes, visible by light microscopy, are not involved. Thirdly, the rate of elimination of the fluorescent label could be much greater in the tumour cells, so that although the rate of uptake may be greater, the amount visible at any time is similar to that seen in normal fibroblasts. It is possible that the experimental conditions did not permit the maximum manifestation of loss of contact inhibition by the sarcoma cells, since, as Curtis (1961) has shown, contact inhibition is affected by the nature of the culture medium and the population density.

When normal epithelial cells were compared with carcinoma cells the results varied too much to allow any simple conclusion to be drawn. Most normal epithelial cells incorporated very small amounts of protein, but all those examined were derived from the kidneys, and epithelia from other organs may behave differently. The carcinomas showed a range of activities. For example, the hamster stilboestrol-induced kidney carcinoma cells showed no more uptake than the normal kidney epithelial cells from which they were presumably derived. The HaK cells, also derived from normal hamster kidneys, had undergone malignant transformation *in vitro* (private communication from Dr. I. M. Spense, Poliomyelitis Research Foundation, Johannesburg) and incorporated considerable quantities of protein (Fig. 8). This raises the issue of the effect of the length of time during which cells have been maintained *in vitro* on the process of protein uptake. Pinocytosis or phagocytosis might confer a nutritional or other advantage, and with cell lines derived after many passages by selection or adaption of cells from primary cultures, an increase in the capacity for protein uptake might occur. It is not possible to decide whether the capacity of HaK cells to incorporate proteins is due solely to the malignant transformation or to an adaptive or selective process not necessarily involving malignancy. The 3rd and 4th passages of rabbit kidney epithelial cells showed no increase in uptake compared with primary cultures of rabbit kidney epithelial cells. Furthermore, HeLa S3 cells which have been maintained in culture for many years incorporate proteins to a very limited extent. This result is in agreement with that which Eagle and Piez (1960) obtained using $^{14}C$-labelled proteins, and with that of Löffler, Henle and Henle (1962) and Rapp (1962), who used the fluorescent antibody techniques and failed to find evidence for the penetration of antibody molecules into HeLa S3 cells. Similarly, the cultures of Fisher lymphosarcoma cells showed active uptake by less than 5 per cent of the population, although these cells had been previously maintained *in vitro* for more than 2 years at this Institute. There seems some support, nevertheless, for the suggestion that in some cases the length of time that the cells have been maintained *in vitro* is responsible for their capacity for pinocytosis or phagocytosis.

The rate of cell multiplication was not by itself sufficient to account for the incorporation of fluorescent protein, although it was observed that cells in some healthy cultures, where there was rapid cell multiplication, incorporated more protein than cells of cultures of the same type which grew much less rapidly. But this increased uptake probably reflects a generally more active metabolism rather than the higher rate of cell division alone. Both the HeLa S3 and Fisher lymphosarcoma cells doubled in number in less than 24 hours, but in spite of this, they incorporated relatively small amounts of protein.

Varying the medium had only slight effects on protein incorporation, provided the medium was capable of supporting the maintenance of the cells. It is difficult to interpret the stimulating effect of tryptose phosphate broth on the protein uptake by C13 cells grown in the presence of Eagle's HeLa medium + 10 per cent calf serum. The rate of cell multiplication certainly increased, but other factors such as motility, adhesion and membrane movements were probably changed also, and it is not clear how far these changes will affect uptake. Removal of serum from the medium did not significantly affect uptake over 24 hours with C13 and TC6 cells, but the presence of the fluorescent proteins may have been an effective substitute for the role, if any, of the serum.

Any differences in the uptake of different proteins by the same cell type appear to be of a minor quantitative nature, in agreement with the observations of Holtzer and Holtzer (1960). Using ferritin, diphtheria toxoid and bovine plasma albumin, no cell type was found which would take up one protein and not the others. The only qualification to this is in the case of a cytotoxic protein. For example, Cormack, Easty and Ambrose (1961) found that a preparation of wheat germ lipase is rapidly pinocytosed by normal hamster kidney cells, which do not pinocytose the three proteins tested in this work to any significant extent. This preparation is now known to contain a phospholipase ; it is cytotoxic, and the pinocytosis which it induces is almost certainly associated with its enzymic action on the cell surface and the cell death which it ultimately produces.

For all the cell types examined, the number and intensity of the fluorescent vacuoles appeared to remain constant after 24–36 hours exposure to any of the three fluorescent proteins. Similarly, all detectable fluorescence was lost from the cells 36–48 hours after removal of the fluorescent proteins from the medium. The methods used in this work did not permit the analysis of the process of uptake. For example, it is not clear whether uptake occurs at a constant or variable rate. The constant level of fluorescence within the cells after 1–2 days was probably due to an equilibrium being reached between elimination and uptake, although the rates of uptake and elimination might vary in a cyclic or irregular manner.

The effect of population density on uptake was tested using C13 fibroblasts only, and cells in the less densely populated cultures appeared to accumulate proteins significantly faster than those in more densely populated cultures (Fig. 13, 14). As with medium effects it is not easy to explain this result. The cells in the less dense population may be capable of more continuous or more rapid movement since cell–cell contacts which temporarily arrest movement should be less frequent, but the relationship between movement, uptake and population density is still largely unknown. It was noted, however, that the cells in the less densely populated cultures possessed a greater surface area than those in the more densely populated cultures, and this increase in exposed surface area might be responsible for the increased uptake. The observation that cells on the periphery of some epithelial islands take up proteins more rapidly than those in the interior suggests that the possession of an actively undulating membrane, accompanied possibly by cell migration, is involved in protein uptake. Similar observations of enhanced peripheral cell phagocytosis have been reported by Bellairs and New (1962) using chick blastoderms *in vitro*.

The uptake of fluorescent bovine plasma albumin by C13 fibroblasts was not affected by $10^{-3}$ M potassium cyanide, indicating that the process was independent of oxidative energy metabolism, at least over a 24-hour period. Sodium fluoride at $2 \times 10^{-2}$ M considerably inhibited uptake, presumably by inhibition of glycolysis. Both these effects are very similar to those obtained by Sbarra and Karnovsky (1959), who investigated the phagocytosis of latex particles by leucocytes. The inhibitory effects of low temperatures on uptake by C13 and TC6 cells emphasizes the dependence of the process on metabolic energy.

Insulin has been reported to stimulate pinocytosis in HeLa cells (Paul, 1959) and adipose tissue cells (Barrnett and Ball, 1960). None of the cells used in this study showed any increased incorporation of fluorescent protein in the presence of insulin, with or without extra glucose, with two exceptions. Mouse mammary carcinoma cells showed slight but consistent increases in uptake in the presence of insulin, and in one of a series of eight experiments, HeLa S3 cells showed a similar stimulation. Insulin has been reported by Prop (1961) to stimulate proliferation of mouse mammary glands *in vitro* and to enable other hormones to exert effects, which suggests that insulin may be acting by facilitating transport of these hormones into the cells, possibly by stimulating pinocytosis. The stimulation of pinocytosis by mouse mammary carcinoma cells treated with insulin may be related to this observation. It is possible that other unknown factors may be involved in this effect. Paul (1959) reported that the stimulating effect on HeLa cells was variable and suggested that the response of the cells to insulin might depend on the treatment which the cells had received before the experiment. Furthermore, although Barrnett and Ball (1960) have shown by electron microscopy that insulin apparently induces numerous invaginations of the plasma membrane in adipose tissue cells, similar work by Orth and Morgan (1962) on myocardium did not reveal any insulin-induced pinocytosis in perfused hearts. The role of insulin in stimulating pinocytosis therefore remains obscure, and the variability of results obtained with cells such as HeLa suggests that other unknown factors are involved, and that insulin by itself is not a universal stimulant of pinocytosis in mammalian cells *in vitro*.

Direct evidence for the presence of the antigenic groups of the ingested protein within cytoplasmic vacuoles was provided by the fluorescent antibody technique using C13 fibroblasts exposed to diphtheria toxoid (Fig. 16). This protein was used because it is completely foreign to the cells and should not cross-react with any indigenous proteins of the cells or the medium. Also, it is one of the few highly purified antigens available whose specific antibody, as opposed to a $\gamma$-globulin fraction, can be obtained in relatively large quantities, possessing a high titre. The retention of nearly all of the fluorescence in the vacuoles of cells fixed in ethyl alcohol, acetone, or 4 per cent formaldehyde indicates that the fluorescent label was still attached to a non-diffusing, high molecular weight component. When fibroblasts were exposed to solutions of sodium fluorescein of comparable fluorescent intensity, the fluorescence visible within the cells was of low intensity and diffuse. Presumably some fluorescein was being pinocytosed but rapidly escaped from the vacuoles. When such cells were fixed after 24 hours exposure to sodium fluorescein, all fluorescence was lost from the cells, indicating that fluorescein was not bound in detectable quantities to any fixable structures within the cells.

The cytology of the process of protein uptake by cells has been described by others in considerable detail (Holtzer and Holtzer, 1960 ; Holter, 1959 ; Chapman-Andresen, 1962), and only certain points will be emphasized here. The fluorescent vacuoles concentrate around the nucleus of the cell, outlining it, but also frequently revealing a clear region adjacent to the nucleus, presumably occupied by the Golgi apparatus (Fig. 5, 6). No diffuse fluorescence due to the protein was detected outside the vacuoles but this may reflect the limits of sensitivity of the technique rather than prove its absence. No sign of incorporation of fluorescence within mitochondria was observed and incorporation within the nucleus in the form of detectable vacuoles, if it occurred at all, was very rare.

Holtzer and Holtzer (1960) have critically reviewed the limitations inherent in the use of fluorescent labelled proteins for examining the uptake of proteins by cells. One of the chief difficulties has been the occurrence of what the Holtzers describe as the " injured cell reaction ". The cytoplasm and nucleus of cells which are permeable to dyes such as lissamine green and trypan blue are stained by fluorescent proteins (Fig. 7), but cells not irreversibly damaged may for a period of time display an altered permeability from which they may later recover. The binding of proteins by damaged cells has been recorded by Ryser *et al.* (1962) and Holtzer and Holtzer (1960), and similar effects have frequently been observed in the work described here. In general, the uptake by cells in any given culture excluding those which are dead and displaying the injured cell reaction, was reasonably uniform from cell to cell. The only consistent exception to this was found with cultures of Fisher lymphosarcoma and Ehrlich's ascites cells, where about 5 per cent of the cells consistently showed considerable uptake in a form not generally associated with cell death. A similar percentage of pinocytosing ascites cells was recorded by Möller and Möller (1962) with three different mouse ascites tumours, and by Holtzer and Holtzer (1960). It is not known whether this small percentage of cells is derived from normal phagocytic cells, or whether they represent a population of tumour cells which differs markedly from the majority in its capacity for protein uptake. A further possibility is that they are deteriorating cells, which Thomason and Schofield (1961) suggested were indulging in much more active membrane movements involving pinocytosis than their healthy counterparts. Whatever the explanation, such cells could give misleading results when methods of measuring uptake are used which assume that the cell population behaves uniformly.

No attempt has been made in this study to distinguish between phagocytosis and pinocytosis. Indeed, it would not be easy to do this, as all cultures contain particles derived from dead cells, secreted extracellular materials and denatured proteins, all of which may bind fluorescent proteins. However, the medium was always renewed 24 hours before the addition of the fluorescent protein and cultures were, in general, free of any gross contamination by particles. Holtzer and Holtzer (1960) have concluded from their work that fibroblasts phagocytose fluorescent particles but are incapable of pinocytosis. Thorough washing of our cultures with centrifuged media and the use of centrifuged fluorescent protein solution free of microscopically visible particles did not result in any decrease in the formation of fluorescent vacuoles within the fibroblasts used in this work. It seems reasonable to assume that the mechanism of uptake involved pinocytosis as well as the phagocytosis of microscopically visible particles.

Staining of plasma membranes was rarely observed, whether or not the cells were washed before examination. With primary embryonic fibroblast cultures, staining of some of the cell surfaces was apparent (Fig. 1, 2) but careful comparison with dark field and phase contrast images revealed that this was generally due to the staining of extracellular material which these cells seem to produce in considerable quantities. This does not mean that no surface absorption takes place but rather that it probably occurs in layers too thin to be detected by the techniques used.

It is suggested from this study that the ability of cells *in vitro* to take up macromolecules from the medium is primarily an inherent function of the particular cell type. Environmental effects seem to be of a second order. Thus the rate of cell division, the nature of the medium or macromolecules present, do not seem to influence to any great extent the ability of the cells to ingest proteins. The basis of these differences in the pinocytotic and phagocytic capacities of different cell types is largely unknown. It is probable, however, that all cells are capable of incorporating macromolecules, at least to a very small extent, which might be detected by more sensitive techniques involving, for example, electron microscopy.

### SUMMARY

(1) The uptake of three fluorescent labelled proteins by monolayer cultures of normal and tumour cells has been investigated using fluorescent microscopy.

(2) Normal fibroblasts and sarcoma cells incorporated the proteins actively and concentrated them as droplets within the cytoplasm. The normal epithelial cells, all derived from the kidneys of various species, did not incorporate significant quantities of protein, whereas the cells of certain carcinomas which had been maintained *in vitro* for long periods were quite active.

(3) Immunofluorescent staining revealed the presence of antigenic groups of one of the proteins within the cytoplasmic vacuoles and therefore indicated the presence of the intact protein.

(4) Cells which incorporated one protein incorporated the other two, and conversely; although there may have been minor quantitative differences in the amounts incorporated.

(5) The effects of variations in the culture medium and the presence or absence of serum were slight, provided the cells remained in a satisfactory condition, showing no morphological deterioration.

(6) Protein uptake by fibroblasts was greater in regions of lower population density and in peripheral cells of islands of epithelia.

(7) Uptake by normal fibroblasts was largely abolished by sodium fluoride, an inhibitor or glycolysis, but not by potassium cyanide, an inhibitor of oxidative metabolism. Uptake was reduced by maintaining the cells at reduced temperatures, indicating the dependence of the process on metabolic energy.

(8) In general, no stimulation of uptake by insulin was observed. Slight stimulation was observed with cultures of mouse mammary carcinoma, and in one out of eight experiments with HeLa S3.

(9) Considerable incorporation was observed in a small proportion (about 5%) of the cells present in both of the ascites tumour cultures examined, but no significant incorporation was observed in the remaining 95 per cent of the cells.

(10) The results indicate that the capacity of cells grown in contact with glass to take up intact macromolecules such as proteins, is inherent in the cell type, and this capacity is to a large extent independent of the environment. Several carcinomas showed considerable uptake but it is not clear whether this was due solely to their malignant nature, or to adaptive and selective processes operating on the cell lines which had been maintained *in vitro* for several years. There is, therefore, no clear correlation between protein uptake and the normal or malignant nature of the cells.

This investigation has been supported by grants to the Chester Beatty Research Institute (Institute of Cancer Research : Royal Cancer Hospital) from the Medical Research Council, the British Empire Cancer Campaign, the Tobacco Research Council and the National Cancer Institute of the National Institutes of Health, U.S. Public Health Service.

### REFERENCES

ABERCROMBIE, M. AND AMBROSE, E. J.—(1958) *Exp. Cell Res.*, **15**, 332.
BARRNETT, R. J. AND BALL, E. G.—(1960) *J. biophys. biochem. Cytol.*, **8**, 83.
BELLAIRS, R. AND NEW, D. A. T.—(1962) *Exp. Cell. Res.*, **26**, 275.
BENNETT, H. S.—(1956) *J. biophys. biochem. Cytol.*, **2**, 99.
CHAPMAN-ANDRESEN, C.—(1962) *C.R. Lab. Carlsberg*, **33**, 73.
CORMACK, D. H., EASTY, G. C. AND AMBROSE, E. J.—(1961) *Nature, Lond.*, **190**, 1207.
CURTIS, A. S. G—(1961) *J. nat. Cancer Inst.*, **26**, 253.

EAGLE, H. AND PIEZ, K. A.—(1960) *J. biol. Chem.*, **235**, 1095.
GEY, G. O.—(1956) *Harvey Lect.*, **50**, 154.
HENDERSON, J. F. AND LEPAGE, G. A.—(1959) *Cancer Res.*, **19**, 887.
HOLTER, H.—(1959) *Int. Rev. Cytol.*, **8**, 481.
HOLTZER, H. AND HOLTZER, S.—(1960) *C.R. Lab. Carlsberg*, **31**, 373.
LEWIS, W. H.—(1935) *Science*, **81**, 545.
LÖFFLER, H., HENLE, G. AND HENLE, W.—(1962) *J. Immunol.*, **88**, 763.
MCCUTCHEON, M., COMAN, D. R. AND MOORE, F. D.—(1948) *Cancer, N.Y.*, **1**, 460.
MERCER, E. H. AND EASTY, G. C.—(1961) *Cancer Res.*, **21**, 52.
MÖLLER, E. AND MÖLLER, G.—(1962) *J. exp. Med.*, **115**, 527.
ORTH, D. N. AND MORGAN, H. E.—(1962) *J. Cell Biol.*, **15**, 505.
PAUL, J.—(1959) *J. exp. Zool.*, **142**, 475.
PROP, F. J. A.—(1961) *Path. Biol., Paris*, **9**, 640.
RAPP, F.—(1962) *J. Immunol.*, **88**, 732.
RIGGS, J. L., SEIWALD, R. J., BURCKHALTER, J., DOWNS, C. M. AND METCALF, T. J.—(1958) *Amer. J. Path.*, **34**, 1081.
RYSER, H., AUB, J. C. AND CAULFIELD, J. B.—(1962) *J. Cell. Biol.*, **15**, 437.
SBARRA, A. J. AND KARNOVSKY, M. L.—(1959) *J. biol. Chem.*, **234**, 1355.
THOMASON, D. AND SCHOFIELD, R.—(1961) *Exp. Cell Res.*, **24**, 457.